# Draft Genome Sequences of Four Strains of *Vibrio parahaemolyticus*, Three of Which Cause Early Mortality Syndrome/Acute Hepatopancreatic Necrosis Disease in Shrimp in China and Thailand

**DOI:** 10.1128/genomeA.00816-14

**Published:** 2014-09-04

**Authors:** Yi-Ting Yang, I-Tung Chen, Chung-Te Lee, Chien-Yu Chen, Shih-Shun Lin, Lien-I Hor, Ta-Chien Tseng, Yun-Tzu Huang, Kallaya Sritunyalucksana, Siripong Thitamadee, Han-Ching Wang, Chu-Fang Lo

**Affiliations:** a Institute of Bioinformatics and Biosignal Transduction, College of Bioscience and Biotechnology, National Cheng Kung University, Tainan, Taiwan; b Department of Life Science, National Taiwan University, Taipei, Taiwan; c Department of Microbiology and Immunology, College of Medicine, National Cheng Kung University, Tainan, Taiwan; d Genome and Systems Biology Degree Program, National Taiwan University and Academia Sinica, Taipei, Taiwan; e Department of Bio-Industrial Mechatronics Engineering, National Taiwan University, Taipei, Taiwan; f Institute of Biotechnology, National Taiwan University, Taipei, Taiwan; g Center of Excellence for Shrimp Molecular Biology and Biotechnology, Faculty of Science, Mahidol University, Bangkok, Thailand; h Department of Biotechnology, Faculty of Science, Mahidol University, Bangkok, Thailand; i Shrimp-Virus Interaction Laboratory, National Center for Genetic Engineering and Biotechnology, National Science and Technology Development Agency, Pathumthani, Thailand; j Institute of Biotechnology, College of Bioscience and Biotechnology, National Cheng Kung University, Tainan, Taiwan; k Center of Bioscience and Biotechnology, National Cheng Kung University, Tainan, Taiwan

## Abstract

We sequenced four *Vibrio parahaemolyticus* strains, three of which caused serious acute hepatopancreatic necrosis disease. Sequence analysis of the virulent strains revealed not only genes related to cholera toxin and the type IV pilus/type IV secretion system but also a unique, previously unreported, large extrachromosomal plasmid that encodes a homolog to the insecticidal *Photorhabdus* insect-related binary toxin PirAB.

## GENOME ANNOUNCEMENT

Early mortality syndrome (EMS), or acute hepatopancreatic necrosis disease (AHPND), is a newly emergent penaeid shrimp disease that causes serious economic losses in shrimp farms. EMS/AHPND is caused only by some pathogenic strains of *Vibrio parahaemolyticus.*

We sequenced three strains of *V. parahaemolyticus* that cause AHPND and one strain that does not. Strains 3HP and 5HP were isolated from the hepatopancreas of EMS/AHPND-diseased *Penaeus vannamei* collected from a shrimp farm on the western coast of the Gulf of Thailand (1). The China strain was isolated in August 2010 from a Hainan Island shrimp pond that showed early signs of mass mortality (1). The non-AHPND strain, S02, was isolated in 2008 from shrimp pond sediment collected from Phang-Nga province, Thailand, before EMS/AHPND emerged (1).

*V. parahaemolyticus* genomic DNA was extracted using a QIAamp DNA minikit (Qiagen), and the paired-end libraries were sequenced by an Illumina MiSeq sequencer. The sequence data were assembled using CLC Genomics Workbench version 7 (Bio CLC), and the contigs were annotated by RAST (2).

The genomes of strains 3HP, 5HP, China, and S02 were assembled into 100, 82, 144, and 97 contigs, respectively, with 5,155, 5,148, 4,950, and 4,946 predicted coding DNA sequences. *V. parahaemolyticus* has two chromosomes: for these four strains, chromosome I was calculated to be approximately 3.360, 3.362, 3.190, and 3.244 Mbp, whereas chromosome II was approximately 1.872, 1.871, 1.755, and 1.927 Mbp, respectively. There were 113, 107, 113, and 102 untranslated RNA sequences, respectively.

Four genes encoding homologues of *V. cholerae* toxins—zona occludens toxin, accessory cholera enterotoxin, transcriptional activator ToxR, and transmembrane regulatory protein ToxS—were found in the 3HP, 5HP, and China strains but not in the S02 strain. The same four genes were also present in an AHPND strain from Mexico (M0605 [3]) and in two of the three AHPND strains from Thailand (TUMSAT_DE1_S1 and TUMSAT_DE2_S2 [4]). However, none of them were found in TUMSAT_D06_S3.

We also found one large (~69 kbp), previously unreported, extrachromosomal plasmid in all three of the AHPND *V. parahaemolyticus* strains but not in the non-AHPND strain. A previously released AHPND PCR detection method (http://www.enaca.org/publications/health/disease-cards/ahpnd-detection-method-announcement.pdf) used primers based on differential sequences, all of which are located on this plasmid (contigs 21 and 61, 4 and 43, and 46 and 13 for 3HP, 5HP, and China, respectively). While most of the open reading frames on this AHPND-associated plasmid are novel genes, we also identified one gene that encodes a homologue of the insecticidal *Photorhabdus* insect-related binary toxin PirAB. Sequence data shows that this AHPND-plasmid protein is a pore-forming protein, but more work will be needed to determine its role in causing the rapid death of the hepatopancreatic cells in shrimp infected with AHPND strains of *V. parahaemolyticus.*

### Nucleotide sequence accession numbers.

The nucleotide sequences of 3HP, 5HP, China, and S02 have been deposited in DDBJ/EMBL/GenBank under accession numbers JPKS00000000, JPKT00000000, JPKU00000000, and JPKV00000000, and named *Vibrio parahaemolyticus* NCKU_TV_3HP, NCKU_TV_5HP, NCKU_CV_CHN, and NCKU_TN_S02, respectively. The versions described here are JPKS01000000, JPKT01000000, JPKU01000000, and JPKV01000000.
